# A Mechanism-Centered Text-Mining Landscape of Mitochondrial Bioenergetics and Signaling in Disease Research

**DOI:** 10.3390/ijms27156577

**Published:** 2026-07-23

**Authors:** Harun Yonar, Furkan Çağrı Beşoluk, Tuğba Melike Parlak

**Affiliations:** 1 Department of Veterinary Biostatistics, Faculty of Veterinary Medicine, Selcuk University, Konya 42130, Türkiye; furkan.besoluk@selcuk.edu.tr; 2 Department of Pharmacology and Toxicology, Faculty of Veterinary Medicine, Selcuk University, Konya 42130, Türkiye; tugba.parlak@selcuk.edu.tr

**Keywords:** mitochondrial bioenergetics, mitochondrial dysfunction, oxidative stress, text mining, non-negative matrix factorization, structural topic modeling, disease–mechanism co-occurrence, mitochondrial therapeutics

## Abstract

Mitochondria are increasingly recognized as integrated bioenergetic and signaling hubs across disease contexts, but the rapidly expanding literature remains fragmented across mechanisms, diseases, and analytical vocabularies. This study mapped the disease-oriented literature on mitochondrial bioenergetics and signaling from 2014 to 2025 using a mechanism-centered text-mining framework integrating dictionary-based annotation and topic modeling. Records retrieved from Web of Science, Scopus, and PubMed were harmonized into a final corpus of 166,462 title–abstract records. Dictionary-based annotation was used to identify disease and mitochondrial mechanism signals, followed by disease–mechanism co-occurrence, lift-based enrichment, exploratory drug/compound annotation, non-negative matrix factorization (NMF), and structural topic modeling (STM). Publication output increased approximately 2.6-fold over the study period. The literature was organized around a central mechanistic backbone involving ROS/redox biology, cell death pathways, bioenergetics/OXPHOS, mitochondrial dysfunction/homeostasis, and quality-control processes. Cancer, cardiometabolic/metabolic disease, and neurodegeneration/neurological injury were the dominant disease contexts. Enrichment analysis revealed disease-characteristic mitochondrial signatures, while NMF identified a 20-topic thematic structure and STM showed increasing emphasis on mitochondrial dysfunction, immune-inflammatory signaling, omics-based prognostic signatures, therapeutic delivery systems, and cancer progression/resistance. Overall, mitochondrial disease research is shifting toward an integrated, application-oriented framework in which mitochondria are positioned as bioenergetic, signaling, immune-regulatory, and therapeutic-response hubs.

## 1. Introduction

Beyond their canonical role in ATP synthesis, mitochondria function as integrative bioenergetic and signaling hubs that coordinate cellular metabolism with redox balance, calcium handling, regulated cell death, innate immune signaling, and stress adaptation. Oxidative phosphorylation through the electron transport chain provides the energetic basis for cellular function, but mitochondrial activity also shapes reactive oxygen species production, metabolite availability, mitochondrial DNA integrity, membrane potential, organelle dynamics, and quality-control pathways such as mitophagy [1,2,3,4]. In this broader view, mitochondria are not passive energy-producing organelles, but dynamic regulatory platforms positioned at the intersection of catabolic and anabolic metabolism, cellular stress adaptation, and metabolic signaling. Their functions are tuned according to tissue-specific energetic, biosynthetic, and repair demands, and mitochondrial signals can influence not only local cellular responses but also organism-level homeostasis [5,6,7].

Mitochondrial signaling is also not restricted to events occurring within the organelle itself. Current frameworks emphasize mitochondrial communication through inter-organelle contacts, vesicular trafficking, and metabolic transmission. Mitochondria–endoplasmic reticulum contact sites, mitochondria-derived vesicles, mitochondrial metabolites, mtDNA-related signals, and quality-control pathways connect mitochondrial function with calcium exchange, lipid handling, immune signaling, proteostasis, stress responses, and cell fate regulation. These communication routes help explain why mitochondrial disruption can propagate energetic failure, oxidative injury, inflammatory activation, and altered cell fate decisions across diverse disease contexts [3,8,9,10].

Mitochondrial dysfunction has therefore emerged as a shared mechanistic feature across a broad spectrum of diseases. Cancer, neurodegenerative disorders, cardiometabolic disease, cardiovascular and renal injury, liver disease, inflammatory disorders, and aging-related conditions differ substantially in clinical phenotype, but they frequently converge on overlapping mitochondrial mechanisms. These include impaired OXPHOS and respiration, excessive ROS and redox imbalance, dysregulated apoptosis and other forms of regulated cell death, altered mitochondrial dynamics, defective mitophagy and quality control, mtDNA instability, calcium dysregulation, and metabolic reprogramming. Importantly, these mechanisms do not operate as isolated pathways; rather, they form interconnected mitochondrial stress, signaling, and adaptation modules whose relative importance may vary across tissues, disease contexts, and experimental models [1,7,11,12,13,14,15,16,17,18].

This convergence is particularly evident in cancer and neurodegenerative research. In cancer, mitochondrial metabolism supports not only ATP production but also biosynthesis, redox balance, metabolic plasticity, tumor microenvironment remodeling, and treatment resistance. In neurodegenerative and aging-related disorders, mitochondrial dysfunction is frequently linked to impaired dynamics, defective mitophagy, altered calcium handling, oxidative stress, inflammatory signaling, and loss of cellular resilience. These disease-specific manifestations further support the need to examine mitochondrial mechanisms as cross-cutting biological themes rather than as isolated disease-specific events [8,19,20,21].

At the same time, the literature on mitochondrial mechanisms in disease has expanded rapidly and become increasingly fragmented across disease areas, experimental models, and mechanistic vocabularies. In the present corpus, the number of records increased from 8396 in 2014 to 21,910 in 2025, corresponding to an approximately 2.6-fold increase in annual publication output. Such expansion makes it difficult for conventional narrative synthesis to capture the structure of the field, especially when mechanistic terms are distributed across cancer biology, neuroscience, metabolism, inflammation, toxicology, pharmacology, and aging research [22].

Existing bibliometric studies, including disease-specific mitochondrial analyses, have often emphasized citation structures, author networks, keyword bursts or disease-specific research trends. These studies have been valuable for documenting field growth and identifying major research hotspots, but fewer studies have jointly mapped disease contexts, mitochondrial mechanism domains, enriched disease–mechanism associations, and temporal topic evolution within a single large-scale corpus. This leaves a gap between disease-specific bibliometric mapping and a broader mechanism-centered synthesis of how mitochondrial bioenergetics and signaling are represented across the disease-oriented literature [13,23,24,25].

Text mining encompasses a broad range of computational approaches for extracting structured information and latent patterns from large textual corpora, including dictionary-based annotation and topic-modeling methods [26,27]. In this study, dictionary-based annotation and unsupervised topic modeling were used as complementary components of an integrated text-mining framework. Dictionary-based annotation provided an interpretable disease–mechanism framework by detecting predefined disease and mitochondrial mechanism categories in title–abstract text. Non-negative matrix factorization was then used to identify latent thematic structures directly from abstracts, whereas structural topic modeling was applied to evaluate how comparable thematic domains changed over time. This combined strategy allowed the study to integrate targeted biological interpretability with data-driven discovery and temporal modeling.

Here, we present a mechanism-centered text-mining landscape of mitochondrial bioenergetics and signaling research in disease contexts from 2014 to 2025, integrating dictionary-based annotation, NMF, and STM within a unified analytical framework. Using 166,462 records retrieved from Web of Science, Scopus, and PubMed, we aimed to: (i) characterize the distribution of major disease contexts and mitochondrial mechanism domains; (ii) identify dominant and enriched disease–mechanism co-occurrence patterns; (iii) define the main latent thematic structure of the abstract corpus using NMF; (iv) evaluate temporal changes in topic prevalence using STM; and (v) explore, as an exploratory translational layer, how drug and bioactive compound categories are co-represented with mitochondrial mechanisms. The goal was not to infer biological causality or therapeutic efficacy, but to map how mitochondrial mechanisms are represented, combined, and temporally reorganized within the disease-oriented literature. The novelty of this study lies not in identifying isolated mitochondrial mechanisms, many of which are already well established, but in systematically mapping their disease-specific co-representation, enriched cross-disease organization, and temporal reconfiguration across a large-scale multi-database corpus.

## 2. Results

The results are presented in seven analytical layers. First, the overall corpus structure was summarized according to the publication year and database source. Second, dictionary-based disease and mechanism annotations were used to characterize the distribution of major disease contexts and mitochondrial mechanism domains, and the accuracy of the dictionary-based annotation framework was evaluated through manual validation. Third, disease–mechanism co-occurrence and enrichment patterns were examined. Fourth, temporal changes in dictionary-based disease and mechanism signals were evaluated. Fifth, an exploratory drug/compound annotation layer was used to provide translational context for intervention-oriented literature signals. Sixth, NMF was used to identify the main latent thematic structure of the abstract corpus. Finally, STM was used to examine the temporal evolution of comparable themes across the 2014–2025 study period.

### 2.1. Corpus Overview

After eligibility filtering, database-source harmonization, and duplicate screening, the final analytical corpus consisted of 166,462 records published between 2014 and 2025. Each record included a document identifier, title, abstract, publication year, and database-source information. Data quality checks showed that all retained records contained valid title, abstract, publication year, and database labels, and all records were within the predefined study period.

Consistent with the analytical design described in the Methods, abstracts were used for NMF- and STM-based topic modeling, whereas concatenated title–abstract text was used for dictionary-based disease, mechanism, and drug/compound annotation. This distinction allowed latent topic models to rely on the richer contextual content of abstracts while preserving title-level disease and mechanism signals for dictionary-based annotation.

Publication output increased markedly over the study period, from 8396 records in 2014 to 21,910 records in 2025, corresponding to an approximately 2.6-fold increase. Annual output increased steadily from 2014 to 2022, followed by a slight decrease in 2023 and renewed growth in 2024 and 2025. The highest annual publication count was observed in 2025. Regarding database-source combinations, the largest subset consisted of records indexed in both Web of Science and Scopus, followed by Scopus-only and Web of Science-only records. Smaller subsets were indexed in combinations involving PubMed.

### 2.2. Result of Dictionary-Based Disease and Mechanism

Dictionary-based annotation was applied to the concatenated title–abstract field to identify explicit disease and mitochondrial mechanism signals across the corpus. Because a single document could contain multiple disease contexts and multiple mitochondrial mechanisms, categories were not treated as mutually exclusive. Therefore, document percentages indicate the proportion of the total corpus in which a given disease or mechanism category was detected.

**Figure 1:**
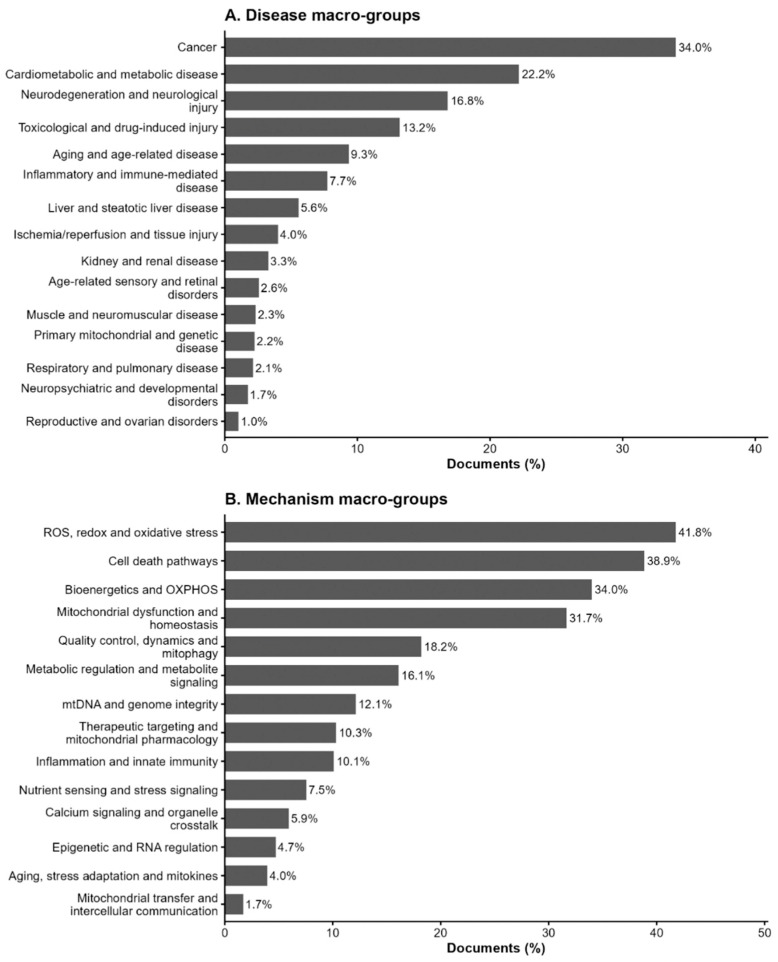
Dictionary-based disease and mechanism annotation of the corpus. (**A**) Disease macro-groups detected in the concatenated title–abstract corpus. (**B**) Mitochondrial mechanism macro-groups detected in the concatenated title–abstract corpus. Percentages indicate the proportion of documents in the total corpus in which each category was detected. Categories are not mutually exclusive.

At the disease level, cancer was the most frequently detected disease macro-group, appearing in 56,592 documents, corresponding to 34.0% of the corpus. Cardiometabolic and metabolic disease was the second most frequent macro-group, detected in 36,944 documents (22.2%), followed by neurodegeneration and neurological injury in 27,992 documents (16.8%). Toxicological and drug-induced injury was identified in 21,947 documents (13.2%), whereas aging and age-related disease appeared in 15,565 documents (9.3%). Other disease macro-groups, including inflammatory and immune-mediated disease, liver and steatotic liver disease, ischemia/reperfusion and tissue injury, kidney and renal disease, respiratory and pulmonary disease, primary mitochondrial and genetic disease, and reproductive and ovarian disorders, were detected at lower but biologically relevant frequencies (Figure 1A).

Among detailed disease categories, cancer was the dominant category, followed by neurodegeneration, diabetes/metabolic disease/obesity, toxicological or drug-induced mitochondrial injury, cardiovascular disease, and aging-related disease. Liver disease and steatotic liver disorders were identified in 9257 documents (5.6%), while ischemia/reperfusion and tissue injury were detected in 6683 documents (4.0%).

At the mechanism level, ROS, redox, and oxidative stress were the most frequently detected mechanism macro-group, appearing in 69,539 documents (41.8%). Cell death pathways were detected in 64,675 documents (38.9%), followed by bioenergetics and OXPHOS in 56,591 documents (34.0%) and mitochondrial dysfunction and homeostasis in 52,710 documents (31.7%). Quality control, dynamics, and mitophagy were identified in 30,313 documents (18.2%), whereas metabolic regulation and metabolite signaling appeared in 26,794 documents (16.1%) (Figure 1B). Additional mechanism macro-groups included mtDNA and genome integrity, therapeutic targeting and mitochondrial pharmacology, inflammation and innate immunity, nutrient-sensing and stress signaling, calcium signaling and organelle crosstalk, epigenetic and RNA regulation, aging/stress adaptation/mitokines, and mitochondrial transfer or intercellular communication.

Detailed mechanism categories further supported this pattern. Oxidative stress/ROS was the most frequent detailed mechanism category, detected in 64,255 documents (38.6%), followed by apoptosis/cell death in 60,103 documents (36.1%), mitochondrial dysfunction in 34,886 documents (21.0%), bioenergetics/respiration/ATP in 26,544 documents (15.9%), mitochondrial function/homeostasis in 25,795 documents (15.5%), redox/antioxidant defense in 24,111 documents (14.5%), and OXPHOS/electron transport chain in 23,566 documents (14.2%).

The multi-label structure of the corpus supported the use of non-mutually exclusive disease and mechanism annotations. Among 166,462 documents, 143,682 documents (86.3%) contained at least one disease label, 152,223 documents (91.4%) contained at least one mechanism label, and 132,493 documents (79.6%) contained both disease and mechanism labels. Documents contained a mean of 1.33 disease labels and 2.75 mechanism labels, with median values of 1 and 3, respectively. These findings indicate that multiple disease and mechanistic contexts frequently co-occurred within individual records.

#### Result of Manual Validation of Dictionary-Based Annotation

Manual validation of the dictionary-based annotation on a stratified sample of 200 records showed high agreement with manual review (Table 1). Micro-averaged precision, recall, and F1-scores were 0.929, 0.931, and 0.930 for disease categories; 0.978, 0.954, and 0.966 for mechanism categories; and 0.888, 0.929, and 0.908 for drug categories, respectively. When true positives, false positives, and false negatives were pooled across all three annotation layers, the overall micro-averaged precision, recall, and F1-score were 0.956, 0.945, and 0.950, respectively. Macro-averaged F1-scores were 0.916 for disease, 0.954 for mechanism, and 0.906 for drug categories, with an overall macro-averaged F1-score of 0.926 across the 77 assessable labels. However, label-level estimates for sparsely represented categories should be interpreted cautiously, as several drug categories were supported by only one or two positive instances in the validation sample, and two drug categories were not represented and therefore could not be independently assessed.

Manual review identified several recurring sources of annotation error. Ambiguous substring matches contributed to false-positive assignments when gene or protein names contained lexical patterns that overlapped with dictionary terms but were unrelated to the intended annotation context. Negation was not consistently captured by the regex-based approach, occasionally producing false-positive annotations when the text explicitly reported the absence of a condition or effect. Broad keyword overlap also reduced precision in selected categories, for example, when generic metabolic terminology triggered disease-related labels or when pathway-level mentions such as mTOR were interpreted as drug-related signals despite the absence of a corresponding pharmacological intervention. These findings indicate that the dictionary framework achieved high overall annotation accuracy while retaining identifiable limitations related to lexical ambiguity and contextual interpretation.

### 2.3. Disease–Mechanism Co-Occurrence and Enrichment

Disease–mechanism co-occurrence analysis was performed to identify how disease macro-groups and mitochondrial mechanism macro-groups were jointly represented within the corpus. Because both disease and mechanism annotations were non-mutually exclusive, co-occurrence values represent documents in which a given disease macro-group and a given mitochondrial mechanism macro-group were detected together. To distinguish corpus-level prominence from disease-specific mechanistic profiles, co-occurrence was evaluated using raw document counts, within-disease percentages, and lift values.

**Figure 2:**
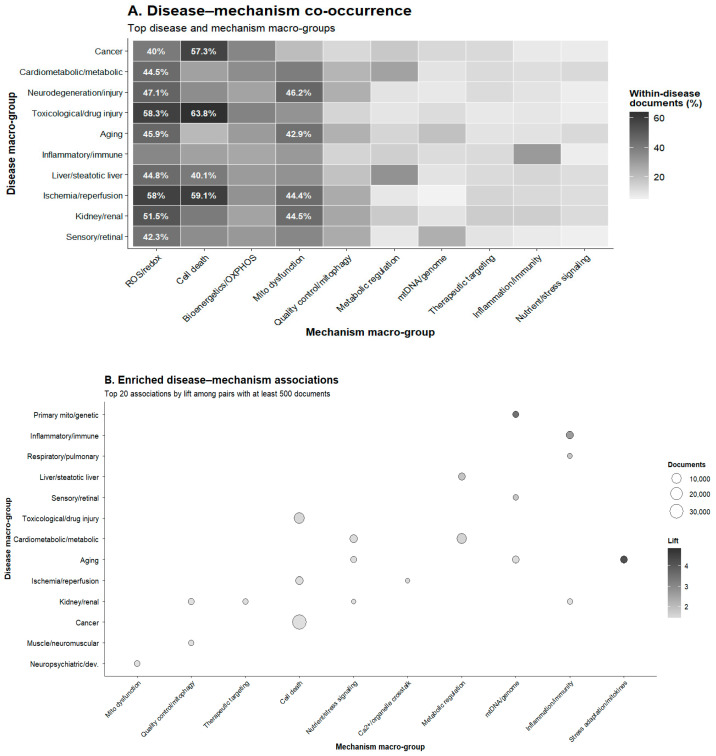
Disease–mechanism co-occurrence and enrichment structure. (**A**) Heatmap showing within-disease co-occurrence percentages between disease macro-groups and mitochondrial mechanism macro-groups. Each cell indicates the percentage of documents within a given disease macro-group in which the corresponding mechanism macro-group was also detected. (**B**) Bubble plot showing the top 20 enriched disease–mechanism associations among pairs with at least 500 co-occurring documents and lift values greater than 1. Bubble size represents the number of co-occurring documents, and color intensity represents lift.

The within-disease heatmap showed that major disease contexts were characterized by distinct but partially overlapping mitochondrial mechanism profiles (Figure 2A). The strongest raw disease–mechanism co-occurrence was observed between cancer and cell death pathways, detected in 32,421 documents, corresponding to 19.5% of the total corpus and 57.3% of the cancer macro-group. Toxicological and drug-induced injury also showed strong co-occurrence with cell death pathways, appearing in 14,007 documents and representing 63.8% of the documents within this disease macro-group. Toxicological and drug-induced injury was additionally associated with ROS, redox, and oxidative stress, which was detected in 12,797 documents and represented 58.3% of this disease group. Ischemia/reperfusion and tissue injury showed a similar injury-centered profile, with high within-disease representation of cell death pathways, ROS/redox biology, and mitochondrial dysfunction/homeostasis. Neurodegeneration and neurological injury were prominently associated with mitochondrial dysfunction and homeostasis, detected in 12,941 documents and representing 46.2% of this disease macro-group. Cardiometabolic and metabolic disease showed a broader metabolic and stress-related profile, including co-occurrence with metabolic regulation and metabolite signaling, ROS/redox biology, mitochondrial dysfunction/homeostasis, and bioenergetics/OXPHOS.

Lift analysis provided a complementary view by identifying disease–mechanism associations that occurred more frequently than expected under independence (Figure 2B). Several enriched pairs showed clear biological coherence. Aging and age-related disease were enriched for aging, stress adaptation, and mitokines. Primary mitochondrial and genetic disease was enriched for mtDNA and genome integrity, with this mechanism detected in 49.1% of documents within the primary mitochondrial and genetic disease macro-group. Inflammatory and immune-mediated disease was enriched for inflammation and innate immunity, whereas liver and steatotic liver disease were enriched for metabolic regulation and metabolite signaling. Cardiometabolic and metabolic disease also showed enrichment for metabolic regulation, and toxicological or drug-induced injury was strongly enriched for cell death pathways.

**Figure 3:**
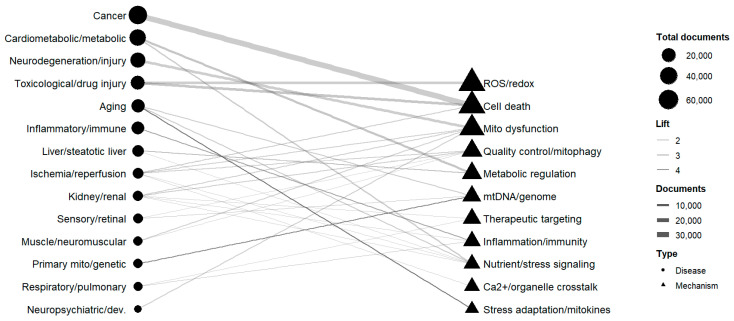
Layered disease–mechanism network based on enriched associations. Disease macro-groups and mitochondrial mechanism macro-groups are shown as separate node types. Edges represent the top 30 enriched disease–mechanism associations among pairs with at least 500 co-occurring documents and lift values greater than 1. Edge width reflects the number of co-occurring documents, whereas edge shading represents lift. The network provides a landscape-level view of disease-specific mitochondrial mechanism enrichment across the corpus.

To provide a landscape-level visualization of enriched disease–mechanism relationships, a layered disease–mechanism network was constructed using lift-based associations (Figure 3). In this network, disease macro-groups and mitochondrial mechanism macro-groups were represented as separate node types, and edges indicated enriched co-detection within the same documents. The network showed that mitochondrial disease research was organized around cross-domain disease–mechanism links rather than isolated disease or mechanism categories. Several biologically coherent patterns were evident, including the connection of primary mitochondrial and genetic disease with mtDNA/genome integrity, inflammatory and immune-mediated disease with inflammation/innate immunity, aging and age-related disease with stress adaptation/mitokines, liver and steatotic liver disease with metabolic regulation, and toxicological or drug-induced injury with cell death pathways.

**Figure 4:**
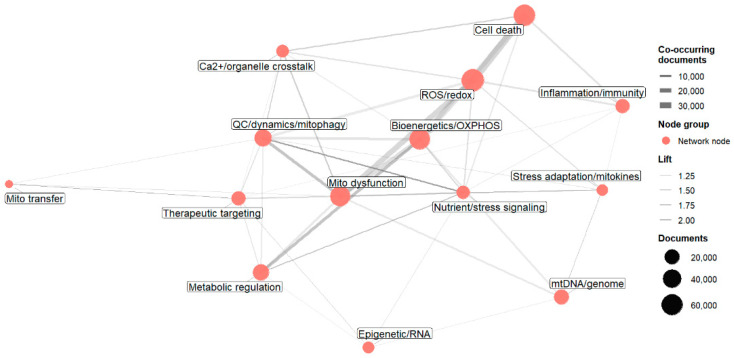
Mechanism macro-group co-occurrence network. Nodes represent mitochondrial mechanism macro-groups, and node size reflects the number of documents in which each mechanism macro-group was detected. Edges indicate co-detection of two mechanism macro-groups within the same documents. Edge width represents the number of co-occurring documents, whereas edge shading represents lift values. The network highlights a central mechanistic backbone involving ROS/redox biology, cell death pathways, bioenergetics/OXPHOS, mitochondrial dysfunction/homeostasis, and quality control/dynamics/mitophagy.

To further examine the internal organization of mitochondrial mechanism categories, a mechanism macro-group co-occurrence network was constructed (Figure 4). This network showed a densely connected mechanistic core centered on ROS/redox biology, cell death pathways, bioenergetics/OXPHOS, mitochondrial dysfunction/homeostasis, and quality control/dynamics/mitophagy. The strongest mechanism–mechanism co-occurrence was observed between cell death pathways and ROS/redox biology, detected in 35,244 documents. Other dominant mechanism pairs included mitochondrial dysfunction/homeostasis with ROS/redox biology, bioenergetics/OXPHOS with ROS/redox biology, bioenergetics/OXPHOS with cell death pathways, and bioenergetics/OXPHOS with mitochondrial dysfunction/homeostasis. Additional mechanism groups, including nutrient/stress signaling, metabolic regulation, therapeutic targeting, calcium/organelle crosstalk, inflammation/innate immunity, and mtDNA/genome integrity, were connected to this core but occupied more peripheral or context-specific positions.

### 2.4. Temporal Dynamics of Dictionary-Based Disease and Mechanism Signals

Temporal analysis of dictionary-based annotations was performed to evaluate how disease and mitochondrial mechanism signals changed across the study period. To improve interpretability, publication years were grouped into three periods: 2014–2017, 2018–2021, and 2022–2025. For each disease and mechanism macro-group, the percentage of documents within each period was calculated, and proportional changes between the earliest and most recent periods were examined.

**Figure 5:**
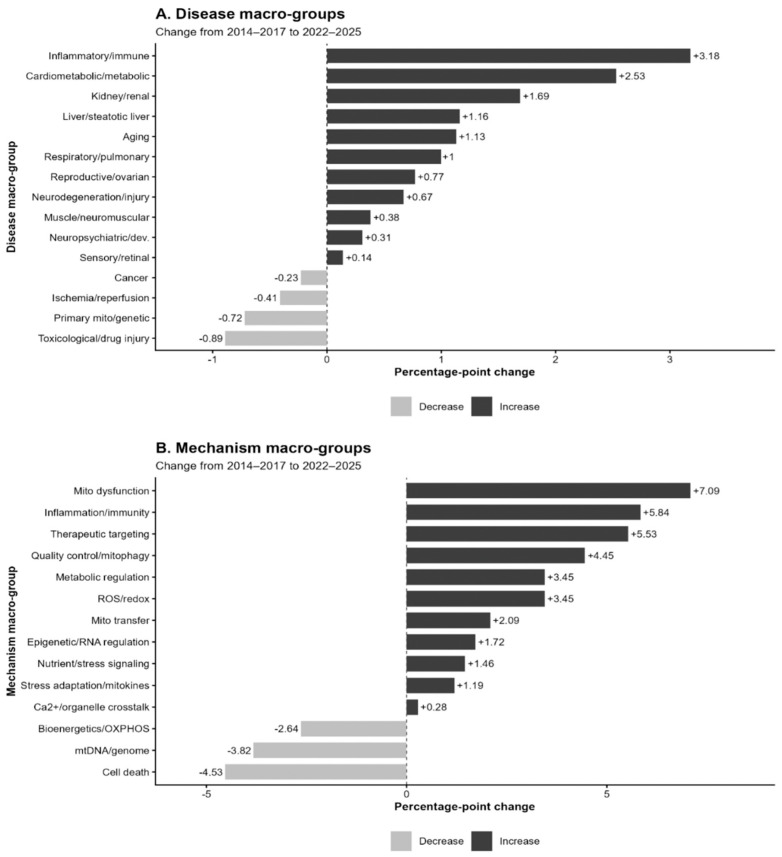
Temporal changes in dictionary-based disease and mitochondrial mechanism signals. (**A**) Absolute percentage-point changes in disease macro-group representation between the early period (2014–2017) and the recent period (2022–2025). (**B**) Absolute percentage-point changes in mitochondrial mechanism macro-group representation between the same periods. Positive values indicate increasing proportional representation in the recent period, whereas negative values indicate decreasing proportional representation. Categories are not mutually exclusive.

At the disease level, inflammatory and immune-mediated disease showed the largest absolute increase, rising from 5.71% in 2014–2017 to 8.89% in 2022–2025, corresponding to a 3.18 percentage-point increase (Figure 5A). Cardiometabolic and metabolic disease also increased, from 20.5% to 23.0%, followed by kidney and renal disease, which increased from 2.20% to 3.89%. Liver and steatotic liver disease increased from 4.87% to 6.03%, and aging and age-related disease increased from 8.77% to 9.90%. Respiratory and pulmonary disease and reproductive and ovarian disorders also showed proportional increases, although they remained lower-frequency disease contexts. In contrast, cancer remained broadly stable across periods, decreasing only slightly from 34.1% to 33.8%. Primary mitochondrial and genetic disease, toxicological and drug-induced injury, and ischemia/reperfusion and tissue injury showed modest decreases in proportional representation.

At the mechanism level, mitochondrial dysfunction and homeostasis showed the largest absolute increase, rising from 28.0% in 2014–2017 to 35.0% in 2022–2025, corresponding to a 7.00 percentage-point increase (Figure 5B). Inflammation and innate immunity increased from 6.72% to 12.6%, and therapeutic targeting and mitochondrial pharmacology increased from 7.17% to 12.7%. Quality control, dynamics, and mitophagy also increased, from 15.3% to 19.8%, while metabolic regulation and metabolite signaling increased from 13.9% to 17.4%. ROS, redox, and oxidative stress remained highly prevalent and increased from 40.2% to 43.6%. Mitochondrial transfer and intercellular communication showed the largest relative increase, rising from 0.48% to 2.57%, although it remained a comparatively lower-frequency category.

In contrast, cell death pathways decreased from 42.4% to 37.8%, mtDNA and genome integrity decreased from 14.7% to 10.9%, and bioenergetics/OXPHOS decreased from 35.4% to 32.7% in proportional representation.

### 2.5. Result of Exploratory Drug/Compound Annotation Layer

**Figure 6:**
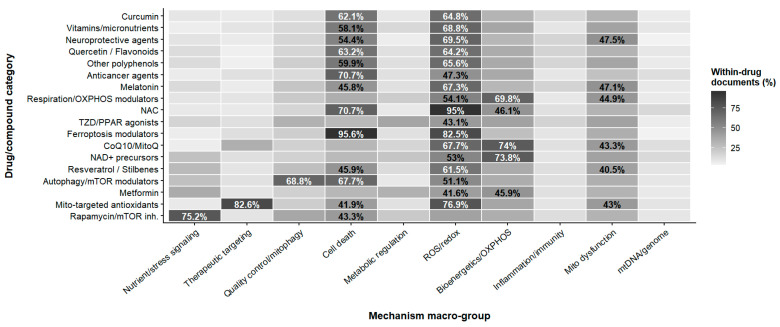
Drug/compound–mechanism co-occurrence structure. Heatmap showing the percentage of documents within each drug/compound category that also contained each mitochondrial mechanism macro-group. Categories are not mutually exclusive. The figure provides an exploratory translational view of how intervention-oriented drug and bioactive compound categories are co-represented with mitochondrial mechanisms in the title–abstract corpus.

To provide additional translational context, an exploratory drug/compound annotation layer was examined in relation to mitochondrial mechanism macro-groups (Figure 6). This layer was not intended to evaluate therapeutic efficacy, but rather to identify how intervention-oriented drug and bioactive compound categories were co-represented with mitochondrial mechanisms within the title–abstract corpus.

Accordingly, the frequency of a drug or compound category reflects only its level of representation in the literature and should not be interpreted as evidence of therapeutic efficacy, clinical effectiveness, comparative benefit, or clinical relevance.

Frequently detected intervention-related categories included vitamins and micronutrients, anticancer targeted agents, natural polyphenols, Coenzyme Q10/ubiquinone/MitoQ, N-acetylcysteine, rapamycin/mTOR inhibitors, and NAD+ precursors. The drug/compound–mechanism heatmap showed that these intervention-oriented categories were primarily co-represented with ROS/redox biology, bioenergetics/OXPHOS, mitochondrial dysfunction/homeostasis, quality control/dynamics/mitophagy, and nutrient-sensing/stress signaling.

Antioxidant- and micronutrient-related categories were strongly connected to ROS/redox biology, whereas NAD+ precursors and respiration/OXPHOS modulators were linked to bioenergetic mechanisms. Rapamycin/mTOR- and autophagy-related categories were associated with nutrient-sensing and mitochondrial quality-control pathways.

### 2.6. NMF-Derived Thematic Structure of the Abstract Corpus

Non-negative matrix factorization was applied to the abstract corpus to identify the main latent thematic structure of mitochondrial bioenergetics and signaling research. Unlike the dictionary-based analyses, which relied on predefined disease and mechanism categories, NMF was used to extract recurring thematic patterns directly from the abstract text. Candidate models with K = 10, 15, 20, 25, and 30 topics were compared using UMass semantic coherence, topic exclusivity, topic-size distribution, and a combined balance score (Table 2).

The 20-topic solution was retained as the final NMF model because it provided the best overall balance between semantic interpretability and topic-size stability. Specifically, K = 20 achieved the highest balance score (0.7891) and the strongest mean UMass coherence among the candidate models (−1.8390), while maintaining acceptable mean exclusivity (0.7185). Although one topic was below 1% based on dominant-topic assignment, the 20-topic solution remained more interpretable than larger models, which showed stronger evidence of topic fragmentation, with two topics below 1% for K = 25 and three topics below 1% for K = 30. Although K = 15 showed slightly higher exclusivity, it had a lower balance score and provided less thematic granularity. Therefore, K = 20 was selected as the most interpretable and stable solution for downstream topic labeling and biological interpretation.

The dominant NMF topic distribution indicated that the abstract corpus was organized around both broad cross-cutting mitochondrial themes and more specific disease- or mechanism-oriented domains, as summarized in Table 3. The largest topic corresponded to aging, cellular homeostasis, and mitochondrial stress-related disorders, accounting for 34,254 documents (20.58%). Other major topics captured cancer metabolic reprogramming and OXPHOS, reproductive and developmental mitochondrial outcomes, mtDNA mutations and mitochondrial genomics, neurodegeneration and neuronal dysfunction, anticancer compounds and cytotoxicity screening, nanoparticle-based cancer therapy and targeted drug delivery, mitochondrial apoptosis and regulated cell death, inflammation and innate immune activation, mitophagy and mitochondrial dynamics, ferroptosis, cardiometabolic disease, liver disease, kidney disease, and skeletal muscle adaptation.

The latest NMF topics were evaluated by considering consistency with the disease–mechanism framework used in dictionary-based analyses, representative documentation, and the highest-ranking terms (Table 3). Several topics directly reflected the dominant mechanism axes identified in the dictionary analysis, including oxidative stress/redox-related biology, apoptosis, and regulated cell death, bioenergetics/OXPHOS, mitochondrial dysfunction, mitophagy, ferroptosis, and mitochondrial biogenesis. Other topics captured disease-centered domains, including cancer metabolism, neurodegeneration, cardiometabolic disease, liver disease, kidney disease, reproductive/developmental outcomes, and skeletal muscle adaptation.

The NMF-derived thematic structure supported and extended the dictionary-based findings. While dictionary annotation showed which predefined disease and mechanism categories were explicitly represented in the corpus, NMF revealed how these signals clustered into broader literature themes.

### 2.7. STM-Based Temporal Evolution of Topics

Structural topic modeling was used to examine how major mitochondrial bioenergetics and signaling themes changed over time across the abstract corpus. After preprocessing, vocabulary filtering, and removal of documents with insufficient remaining tokens, the final STM corpus included 165,324 documents and 4662 terms. A 20-topic STM model was fitted to maintain comparable thematic granularity with the final NMF solution and to support temporal interpretation of topic prevalence.

Although both NMF and STM were fitted with 20 topics, the resulting topics were not interpreted as one-to-one equivalents. NMF was used to identify the main static thematic structure of the abstract corpus, whereas STM was used to examine temporal variations in related thematic domains. Therefore, the two models were integrated at the level of broader biological themes rather than by direct topic-number matching.

The STM solution produced biologically interpretable topics that were broadly consistent with the NMF-derived thematic structure, while adding a temporal dimension to the analysis. The topics captured several disease- and mechanism-related domains, including neurodegeneration and mitochondrial dysfunction, anticancer compound screening, mitochondrial energy metabolism and OXPHOS, cancer progression and resistance, calcium signaling and membrane homeostasis, nanoparticle-based drug delivery and imaging, cardiac and renal injury, apoptosis and autophagy, mtDNA mutations and mitochondrial genetic disorders, inflammation and immune responses, oxidative stress, muscle-metabolic disease, and liver-related mitochondrial metabolism.

**Figure 7:**
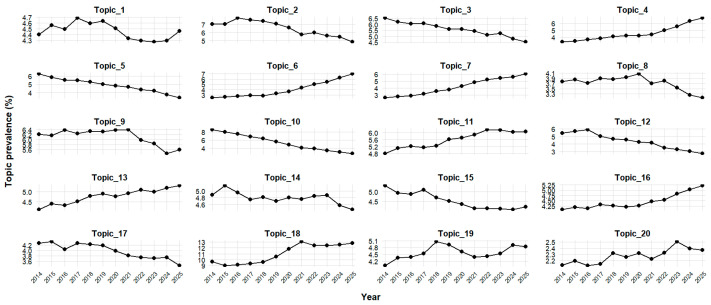
Selected temporal trajectories of STM-derived topics. Annual mean topic prevalence was calculated from document-level topic proportions in the final 20-topic STM model. The figure shows the ten topics with the largest absolute year slopes, representing the strongest increasing and decreasing temporal patterns across the 2014–2025 study period. Topic labels were assigned based on highest-probability, FREX, lift, and score terms together with representative documents.

For temporal interpretation, the annual mean topic prevalence was calculated from document-level STM topic proportions. Topic-level year slopes were used as screening measures to identify the strongest increasing and decreasing temporal patterns. The ten STM topics with the largest absolute year slopes are summarized in Table 4 and visualized in Figure 7.

The strongest increasing temporal signals were observed for gene-expression, transcriptomic, and prognostic signature topics, review-oriented neurodegeneration and mitochondrial dysfunction, inflammation and immune responses, nanoparticle-based drug delivery and imaging, and cancer progression/resistance. These increasing topics suggest that recent mitochondrial disease research has become more strongly oriented toward omics-based stratification, translational delivery systems, immune-inflammatory mechanisms, and disease-specific mitochondrial dysfunction.

In contrast, decreasing relative topic prevalence was observed for apoptosis/autophagy and BCL-2/BAX-mediated cell death, mtDNA mutations and mitochondrial genetic disorders, anticancer compound and cytotoxicity screening, calcium signaling and membrane homeostasis, and selected DNA sequence/species-level genetic content.

The selected temporal trajectory plot confirmed these divergent patterns (Figure 7). Increasing trajectories were most evident for topics related to transcriptomic and prognostic signatures, neurodegeneration-oriented mitochondrial dysfunction, inflammation and immune responses, nanoparticle-based delivery, and cancer progression/resistance. Decreasing trajectories were most evident for apoptosis/autophagy and BCL-2/BAX-mediated cell death, mtDNA/genetic disease, anticancer cytotoxicity screening, and calcium/NLRP3-related signaling. The DNA sequence/species-level topic also showed a decreasing trajectory and should be interpreted cautiously as a sequence- or comparative-genetic literature signal rather than a direct disease–mechanism theme.

A sensitivity analysis comparing K = 15 and K = 20 STM solutions was performed on a random subset of 40,000 documents. Both candidate models produced acceptable topic-size distributions, with no topics below 1% prevalence. The K = 15 model showed slightly greater compactness, whereas the K = 20 model provided more detailed thematic resolution and remained consistent with the final NMF topic granularity. Therefore, K = 20 was retained for the final STM analysis because it provided sufficient temporal interpretability without evidence of excessive topic fragmentation.

Recent years showed increasing attention to mitochondrial dysfunction, immune-inflammatory signaling, omics-based prognostic signatures, therapeutic delivery systems, cancer progression/resistance, and neurodegeneration-oriented translational themes. In contrast, earlier years were relatively more dominated by mtDNA/genetic, calcium signaling, cell death, and compound-screening topics.

Table 4 summarizes the ten STM topics with the largest absolute year slopes in the final 20-topic STM model. Prevalence indicates estimated overall topic prevalence in the STM corpus. Trend direction was based on topic-level year slopes and annual mean topic prevalence trajectories. Topic labels were assigned using highest-probability, FREX, lift, and score terms together with representative documents.

## 3. Discussion

This study provides a mechanism-centered landscape of mitochondrial bioenergetics and signaling research in disease contexts from 2014 to 2025. By combining dictionary-based disease–mechanism annotation, co-occurrence and lift-based enrichment analyses, as well as NMF-derived thematic modeling and STM-based temporal modeling, this study maps not only how frequently mitochondrial mechanisms are represented in the literature, but also how they are organized across disease areas and how their thematic emphasis has changed over time. This combined approach is consistent with the increasing use of text-mining methods, including topic modeling, to synthesize large and fragmented biomedical studies [26,27,28]. The findings indicate that mitochondrial disease research is structured around a central mechanistic backbone involving ROS/redox biology, cell death pathways, bioenergetics/OXPHOS, mitochondrial dysfunction/homeostasis, and quality-control processes, while recent literature increasingly emphasizes inflammation, therapeutic targeting, mitochondrial homeostasis, omics-based signatures, and delivery systems for clinical translation.

The approximately 2.6-fold increase in annual publication output, from 8396 records in 2014 to 21,910 records in 2025, confirms the expanding role of mitochondria in disease-oriented biomedical research. This growth reflects a broader conceptual shift in mitochondrial biology. Mitochondria are no longer viewed only as ATP-producing organelles, but as dynamic bioenergetic and signaling platforms that regulate redox balance, calcium handling, organelle communication, innate immune signaling, metabolic adaptation, quality control, and cell fate [1,2,3,4,11,29,30,31,32]. The dominance of ROS/redox/oxidative stress, cell death pathways, bioenergetics/OXPHOS, and mitochondrial dysfunction/homeostasis in the present corpus supports this integrative view. These mechanisms appear to represent the common language through which diverse disease fields describe mitochondrial involvement [2].

At the disease level, cancer was the most prominent disease macro-group, followed by cardiometabolic/metabolic disease and neurodegeneration/neurological injury. This distribution is biologically coherent. In cancer, mitochondrial metabolism supports bioenergetic flexibility, biosynthesis, redox buffering, apoptosis resistance, tumor progression, and treatment response [13,19,33,34,35]. In neurodegenerative disease, mitochondrial dysfunction is closely linked to neuronal energy failure, oxidative stress, impaired mitophagy, calcium dysregulation, and cellular vulnerability [1,14,18,24]. In cardiometabolic disease, mitochondrial mechanisms are strongly connected to substrate utilization, lipid metabolism, insulin resistance, inflammatory stress, and tissue-specific energetic remodeling [16,23,36,37]. Thus, the dominant disease categories identified in this study reflect major areas in which mitochondrial bioenergetics and signaling have become central mechanistic frameworks.

A major strength of the present analysis is the distinction between frequent co-occurrence and enriched disease–mechanism association. Raw co-occurrence identified highly represented disease–mechanism pairs, such as cancer with cell death pathways, but lift analysis revealed which pairs occurred more often than expected under independence. This distinction is important because the most frequent pairs are not necessarily the most disease-specific. Enrichment analysis showed biologically meaningful associations: primary mitochondrial and genetic disease were enriched for mtDNA and genome integrity; inflammatory and immune-mediated disease for inflammation and innate immunity; liver and steatotic liver disease for metabolic regulation and metabolite signaling; aging-related disease for stress adaptation and mitokine-related mechanisms; and toxicological or drug-induced injury for cell death and ROS/redox biology. These disease–mechanism patterns are consistent with established roles of mitochondrial genetics in primary mitochondrial disorders, mitochondrial immune signaling in inflammation and infection, metabolic remodeling in liver and cardiometabolic disease, and mitochondrial stress responses in aging-related pathology [8,19,20,21,34]. Overall, these findings indicate that mitochondrial mechanisms are not distributed randomly across disease areas, but form disease-characteristic mechanistic signatures.

The mechanism co-occurrence network further supports a systems-level interpretation of mitochondrial dysfunction. ROS/redox biology, cell death pathways, bioenergetics/OXPHOS, mitochondrial dysfunction/homeostasis, and quality control/dynamics/mitophagy formed a densely connected core. This structure is biologically expected because these processes are functionally interdependent. Defective electron transport can increase ROS production; excessive ROS can damage mitochondrial proteins, lipids, and DNA; mitochondrial damage can activate mitophagy, apoptosis, or inflammatory signaling; and impaired quality control can amplify energetic failure and stress responses [3,12,38,39,40,41]. Therefore, the network does not simply describe term co-occurrence; it reflects the conceptual organization of mitochondrial disease biology around interacting stress, signaling, energetic, and cell fate modules.

Temporal analysis showed that the field is not only expanding, but also changing in emphasis. Mitochondrial dysfunction/homeostasis showed the largest increase among mechanism macro-groups, suggesting that recent studies increasingly frame mitochondrial involvement as a problem of integrated organelle homeostasis rather than as a single isolated defect. Inflammation and innate immunity also increased substantially, consistent with growing interest in immunometabolism, inflammasome activation, mitochondrial danger signals, and mtDNA-mediated immune responses [10,42,43,44]. Interventional and mechanistic pharmacology directed at mitochondrial targets increased as well, indicating a stronger clinical translation orientation. In contrast, cell death pathways, mtDNA/genome integrity, and bioenergetics/OXPHOS showed decreasing proportional representation, although they remained highly prevalent. This pattern should not be interpreted as declining biological importance. Rather, it suggests that classical mitochondrial themes are now being embedded within broader frameworks involving immune signaling, quality control, disease progression, omics-based stratification, and therapeutic modulation.

The NMF analysis confirmed and extended the dictionary-based findings by identifying the latent thematic architecture of the abstract corpus. The final 20-topic solution captured both broad cross-cutting themes and disease- or mechanism-specific domains, including aging and mitochondrial homeostasis, cancer metabolism, neurodegeneration, apoptosis, inflammation, mitophagy, ferroptosis, cardiometabolic disease, liver disease, kidney disease, skeletal muscle adaptation, and cancer-directed drug/nanoparticle research. The use of NMF is appropriate in this context because it provides an interpretable matrix factorization framework for extracting non-negative latent components from high-dimensional biomedical text data [27,45,46]. Importantly, NMF did not merely reproduce the predefined dictionaries. Instead, it showed how mitochondrial mechanisms and disease contexts clustered into recognizable research themes. The separation of cancer-related topics into cancer metabolism/OXPHOS, anticancer screening/cytotoxicity, and nanoparticle-based drug delivery is particularly informative, suggesting that mitochondrial cancer research has diversified into metabolic, experimental, and applied-delivery branches.

Several NMF-derived topics also reflected emerging or specialized mechanistic areas. The mitophagy and mitochondrial quality-control topic was characterized by PINK1, Parkin, LC3, fission, and autophagy-related vocabulary, consistent with the established role of selective mitochondrial clearance in mitochondrial homeostasis and disease adaptation [39,40,41]. The ferroptosis topic, although smaller in size, was biologically meaningful because it captured vocabulary related to iron, GPX4, glutathione, lipid peroxidation, and NRF2, aligning with the role of ferroptosis as a regulated cell death modality linking metabolism, redox biology, and disease [47,48]. Similarly, topics related to omics, prognostic immune signatures, and non-coding RNA regulation indicate that mitochondrial disease research increasingly intersects with molecular profiling and systems-level interpretation.

STM added a complementary temporal dimension to this thematic structure. Structural topic modeling is particularly useful for this study because it allows topic prevalence to be modeled in relation to covariates such as the publication year and database source [28,49]. Increasing STM topics included gene-expression/transcriptomic and prognostic signatures, neurodegeneration-oriented mitochondrial dysfunction, inflammation and immune responses, nanoparticle-based delivery and imaging, and cancer progression/resistance. These trends indicate that recent mitochondrial disease research is moving toward data-rich molecular profiling, disease stratification, immune-inflammatory mechanisms, and applied intervention strategies [10,24,49,50]. Conversely, decreasing relative prevalence was observed for apoptosis/autophagy, mtDNA mutations and mitochondrial genetic disorders, anticancer cytotoxicity screening, and calcium signaling/membrane homeostasis. These topics remain biologically important, but their relative decline suggests that the field has shifted from classical pathway-centered and screening-oriented studies toward more integrated and clinically oriented frameworks.

The exploratory drug/compound annotation layer further supports this shift toward application. Intervention-related categories such as vitamins and micronutrients, natural polyphenols, Coenzyme Q10/ubiquinone/MitoQ, N-acetylcysteine, rapamycin/mTOR inhibitors, NAD+ precursors, and anticancer targeted agents were mainly co-represented with ROS/redox biology, bioenergetics/OXPHOS, mitochondrial dysfunction/homeostasis, quality control/dynamics/mitophagy, and nutrient-sensing/stress signaling. These patterns are consistent with current interest in mitochondria-targeted antioxidants, NAD+ metabolism, autophagy modulation, mitochondrial quality control, and nanoparticle-based delivery approaches [19,41,50]. However, these findings should be interpreted strictly as literature-level co-annotation patterns and indicators of research attention, not as evidence of therapeutic efficacy, clinical effectiveness, comparative benefit, treatment recommendation, or clinical relevance. Higher annotation frequency may simply reflect greater research activity or broader use of a compound class in experimental studies and should not be interpreted as having superior therapeutic value. The analysis therefore does not establish causality, drug action, pharmacological effectiveness, or clinical benefit. Accordingly, the drug/compound layer should be regarded as an exploratory translational extension rather than a systematic pharmacological or therapeutic efficacy analysis.

Methodologically, the combined analytical strategy offers advantages over a single bibliometric or topic-modeling approach. Dictionary-based annotation provided biological interpretability and enabled explicit disease–mechanism mapping. Lift analysis identified enriched disease-specific associations rather than only frequent terms. NMF extracted latent thematic domains directly from abstracts, while STM modeled temporal changes in topic prevalence. The convergence of these independent layers strengthens the overall interpretation: mitochondrial disease research is organized around a stable mechanistic core, but its recent direction is increasingly defined by inflammation, mitochondrial homeostasis, therapeutic targeting, omics-based signatures, and application-oriented delivery technologies. This multi-layered strategy is consistent with previous arguments that large-scale text mining, including topic-modeling approaches, can support systematic synthesis when conventional narrative review is insufficient for rapidly expanding studies [22,26,27,51].

Several limitations should be acknowledged. First, the analysis was based on titles and abstracts rather than full-text articles. Abstracts capture major disease and mechanism signals, but may omit experimental details, negative findings, and mechanistic nuance. Second, dictionary-based annotation depends on predefined terms and regular expression patterns. Although manual validation on a stratified sample of 200 records indicated high overall annotation accuracy (pooled micro-averaged F1 = 0.950; overall macro-averaged F1 = 0.926), performance was not uniform across all categories. Sparsely represented drug categories were associated with greater uncertainty, and two drug categories were not represented in the validation sample and therefore could not be independently assessed. Manual review indicated that the remaining errors were mainly related to ambiguous substring matches, unparsed negation, and broad keyword overlap. Accordingly, the dictionary-based framework should be interpreted as a scalable approach for detecting literature-level co-annotation patterns rather than as a fully validated biomedical entity-recognition or diagnostic classification system, and category-specific prevalence may be modestly over- or underestimated. Drug/compound annotation frequencies should likewise be interpreted only as indicators of literature-level representation and not as measures of therapeutic efficacy or clinical relevance. In addition, the study-specific dictionaries were not formally mapped to controlled biomedical vocabularies such as MeSH, UMLS, or GO; future ontology-based mapping could further improve terminology standardization and cross-resource interoperability. Third, co-occurrence and lift analyses indicate literature-level co-representation, not biological causality or experimentally validated mechanistic relationships. Fourth, NMF and STM are sensitive to preprocessing choices, vocabulary filtering, and topic-number selection. Although model selection metrics and interpretability checks supported the final solutions, alternative specifications may produce somewhat different topic boundaries [45,46,49].

Fifth, the retrieval strategy was designed to capture mitochondrial bioenergetics and signaling mechanisms across disease-related biomedical literature rather than to exhaustively represent any single disease domain. As a result, the corpus may overrepresent disease contexts and mechanistic vocabularies that explicitly use mitochondrial signaling terminology, while underrepresenting clinical or translational studies that discuss disease processes without explicit mitochondrial mechanism terms. Sixth, the corpus was restricted to English-language records, which may under-represent research published in other languages and introduce linguistic or regional bias. Seventh, the observed 2.6-fold increase in publication output may partly reflect database coverage expansion, indexing changes, or changes in publication practices rather than purely organic growth of the field. Although the database source was included as an adjustment covariate in the STM specification, residual influence from database coverage and indexing variations cannot be fully excluded. Finally, some NMF- and STM-derived topics, such as anticancer cytotoxicity screening or experimental validation methods, may reflect recurring experimental designs or methodological vocabularies rather than distinct disease biology; this should be considered when interpreting topic-level trends as biological signals.

Despite these limitations, this study provides a large-scale, biologically interpretable and temporally informed map of mitochondrial bioenergetics and signaling in disease research. The findings show that the field is moving from isolated pathway-centered descriptions toward a more integrated view of mitochondria as bioenergetic, signaling, immune-regulatory, and therapeutic-response hubs. This mechanism-centered landscape may help researchers identify dominant research axes, disease-specific mitochondrial mechanism profiles, emerging application-oriented directions, and areas requiring more focused mechanistic or systematic synthesis.

## 4. Materials and Methods

### 4.1. Study Design and Analytical Workflow

This study was designed as an integrated mechanism-centered text-mining analysis combining title–abstract dictionary-based annotation with abstract-based topic modeling of mitochondrial bioenergetics and signaling research in disease contexts. The analytical framework was developed to characterize the distribution of disease-related mitochondrial mechanisms, identify dominant disease–mechanism co-occurrence patterns, extract latent thematic structures from the literature, and evaluate their temporal evolution between 2014 and 2025.

**Figure 8:**
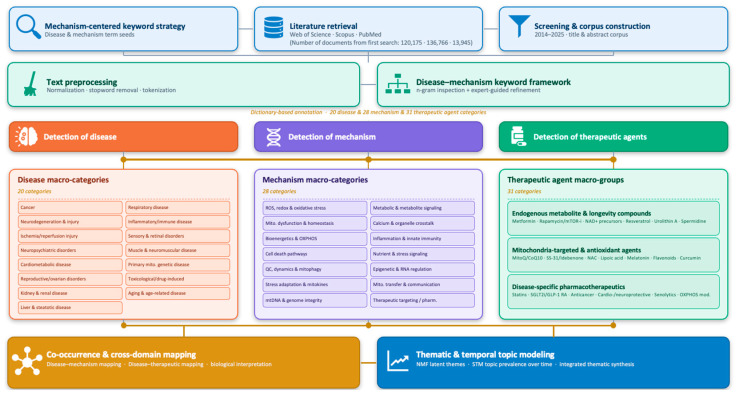
Analytical workflow of the study.

The workflow consisted of seven main stages: literature retrieval using a biologically informed mechanism-centered keyword strategy; corpus construction and preprocessing; exploratory unigram, bigram, and trigram inspections; development of disease and mitochondrial mechanism dictionaries; dictionary-based disease–mechanism annotation; NMF-based thematic modeling; and STM-based temporal topic modeling (Figure 8).

After the dictionary framework and matching rules had been finalized, the accuracy of the dictionary-based annotation approach was additionally evaluated through a separate manual validation procedure.

### 4.2. Data Sources and Literature Retrieval

Scientific records were retrieved from Web of Science, Scopus, and PubMed using a mechanism-centered keyword strategy designed to capture literature related to mitochondrial bioenergetics and signaling in disease-oriented biomedical research. The retrieval strategy focused on core and emerging mitochondrial domains, including mitochondrial bioenergetics, mitochondrial dysfunction, mitochondrial signaling, mitochondrial metabolism, mitochondrial homeostasis, mitochondrial quality control, mitochondrial biogenesis, oxidative phosphorylation, mitochondrial dynamics, mitochondrial fission and fusion, mitophagy, mitochondrial DNA, mitochondrial membrane potential, electron transport chain activity, reactive oxygen species, oxidative stress, calcium signaling, redox homeostasis, ATP production, apoptosis, inflammation, ferroptosis, metabolic reprogramming, cell death, endoplasmic reticulum stress, NAD+ metabolism, and mitochondrial therapeutic targeting.

A representative Boolean search structure was used and adapted to the field tags and search syntax of each database: (mitochondria OR mitochondrial OR mitochondrion OR “mitochondrial bioenergetics” OR “mitochondrial dysfunction” OR “mitochondrial signaling” OR “mitochondrial metabolism” OR “mitochondrial homeostasis” OR “mitochondrial quality control” OR “mitochondrial biogenesis” OR “oxidative phosphorylation” OR OXPHOS OR “electron transport chain” OR “mitochondrial respiration” OR “mitochondrial dynamics” OR “mitochondrial fission” OR “mitochondrial fusion” OR mitophagy OR “mitochondrial DNA” OR mtDNA OR “mitochondrial membrane potential” OR “reactive oxygen species” OR ROS OR “oxidative stress” OR “redox homeostasis” OR “calcium signaling” OR apoptosis OR “regulated cell death” OR ferroptosis OR pyroptosis OR necroptosis OR inflammation OR “innate immunity” OR “metabolic reprogramming” OR “fatty acid oxidation” OR “endoplasmic reticulum stress” OR “NAD+ metabolism” OR “mitochondrial therapeutic targeting”).

The query was applied to title, abstract, and keyword fields where available. Database-specific syntax was adapted for Web of Science, Scopus, and PubMed [52,53,54]. Records were restricted to English review and research article publications from 2014 to 2025. Only records with a valid title, abstract, publication year, and database-source information were retained. The retrieval strategy was designed to capture mitochondrial bioenergetics and signaling mechanisms across disease-related biomedical literature rather than to restrict the corpus to predefined disease names. Disease contexts were subsequently identified using the dictionary-based annotation framework described below.

### 4.3. Corpus Construction

All retrieved records were merged into a unified dataset and screened for eligibility. Records were retained if they contained a valid document identifier, title, abstract, publication year, and database-source information. Publication years were converted to numeric format and checked against the predefined 2014–2025 study period.

Records were harmonized across databases using normalized article titles and database-source metadata. Database-source combinations were retained to indicate whether a record was retrieved from a single database or from overlapping database sources. Duplicate screening and eligibility filtering were performed before defining the final analytical corpus.

Two textual representations were prepared for complementary analytical purposes. Abstracts were used as the primary textual corpus for NMF-based and STM-based topic modeling because they provide richer contextual and mechanistic information for latent theme extraction. Titles and abstracts were concatenated into a single text field for dictionary-based disease, mechanism, and drug/compound annotation, as well as exploratory n-gram inspection. This title–abstract representation was used to improve the detection of explicit disease names, mitochondrial mechanisms, and pathway-related terms that may appear either in article titles or abstracts.

### 4.4. Text Preprocessing

Text preprocessing was performed before dictionary-based annotation and topic modeling. All text fields were converted to character format and transformed to lowercase. Greek symbols and selected biomedical expressions were normalized to improve lexical consistency. Non-informative symbols, excessive whitespace, web addresses, copyright statements, and other irrelevant textual artifacts were removed where applicable. Standard English stopwords and study-specific custom stopwords were excluded to reduce the influence of high-frequency but semantically uninformative terms.

Selected multi-word biomedical expressions were preserved as compound terms before tokenization, including oxidative stress, reactive oxygen species, oxidative phosphorylation, electron transport chain, mitochondrial dysfunction, mitochondrial dynamics, mitochondrial membrane potential, mitochondrial quality control, mitochondrial biogenesis, cell death, calcium signaling, fatty acid oxidation, metabolic reprogramming, and related disease-specific expressions. For NMF, a TF–IDF-weighted document–term matrix was constructed from the cleaned abstract corpus. For STM, a structured document–term representation was prepared from abstracts while retaining publication year and database-source metadata for temporal modeling.

### 4.5. Development of the Disease–Mechanism Keyword Framework

After corpus construction, disease- and mechanism-related terms were refined through exploratory unigram, bigram, and trigram inspection together with expert-guided evaluation of mitochondrial disease biology. This step was used to support the construction of a disease–mechanism keyword framework rather than to generate standalone descriptive findings.

The final disease dictionary included 20 detailed disease categories covering cancer; neurodegeneration; neurological injury and stroke/trauma; ischemia/reperfusion and tissue injury; neurodevelopmental and psychiatric disorders; diabetes/metabolic disease/obesity; reproductive and ovarian disorders; cardiovascular disease; kidney disease; liver and steatotic liver disorders; respiratory and pulmonary disease; sepsis and inflammatory disease; infectious, viral and parasitic disease; autoimmune and immune-mediated disease; ophthalmic and retinal disease; hearing and auditory disorders; skeletal muscle and neuromuscular disease; primary mitochondrial and genetic disease; toxicological or drug-induced mitochondrial injury; and aging-related disease.

The final mitochondrial mechanism dictionary included 28 detailed categories covering oxidative stress and ROS, redox and antioxidant defense, mitochondrial function and homeostasis, mitochondrial dysfunction, membrane potential, mitochondrial permeability transition, apoptosis and regulated cell death, ferroptosis, pyroptosis and necroptosis, mitophagy and autophagy, mitochondrial dynamics, mitochondrial biogenesis and quality control, mitohormesis and mitokines, mtDNA integrity, DNA damage and repair, OXPHOS and electron transport chain activity, bioenergetics and respiration, metabolic regulation and metabolite signaling, fatty acid oxidation and lipid metabolism, mitochondrial calcium signaling, ER stress, inflammatory and innate immune signaling, senescence, AMPK/mTOR/Sirtuin/Sestrin signaling, epigenetic and non-coding RNA regulation, extracellular vesicles, mitochondrial transfer, and therapeutic targeting.

Detailed categories were used for computational annotation, whereas broader disease and mechanism macro-groups were used for main-text visualization and biological interpretation.

The disease and mitochondrial mechanism dictionaries were developed through an iterative, expert-guided process combining domain knowledge in mitochondrial and disease biology with corpus-driven inspection of frequent unigram, bigram, and trigram patterns. Initial candidate terms were identified from recurrent expressions observed in the corpus and from established biomedical terminology commonly used to describe the corresponding disease and mitochondrial mechanism domains. Candidate terms were subsequently expanded to include commonly used synonyms, abbreviations, spelling variants, and closely related phrase-level expressions when these forms referred to the same intended biomedical concept.

Candidate terms were retained when they demonstrated clear biological relevance to the predefined category, sufficient lexical specificity for title–abstract annotation, and an interpretable relationship with the intended disease or mitochondrial mechanism concept. Terms were excluded or modified when they were overly broad, frequently used outside the intended biological context, produced recurrent false-positive matches, or represented ambiguous abbreviations or substrings with multiple unrelated biomedical meanings. Ambiguous terms were refined using more specific phrase-level expressions, word-boundary constraints, and bounded regular expression patterns where appropriate. Dictionary refinement was iterative during the development stage, with candidate matches inspected and problematic terms revised or removed when recurrent misclassification patterns were identified. After the dictionaries and matching rules had been finalized, they were held fixed for the subsequent manual validation procedure described in Section Manual Validation of Dictionary-Based Annotation of Section 4.6.

The dictionaries were developed as study-specific, corpus-informed lexicons and were not directly derived from or formally mapped to controlled vocabularies such as Medical Subject Headings (MeSH), the Unified Medical Language System (UMLS), or Gene Ontology (GO). These resources were therefore not used as formal ontology-based annotation systems in the present analysis. Instead, the framework prioritized corpus-specific lexical coverage, domain-informed term selection, iterative ambiguity resolution, and subsequent manual performance validation.

### 4.6. Dictionary-Based Disease and Mechanism

Dictionary-based annotation was performed to identify explicit disease- and mechanism-related signals within the concatenated title–abstract corpus. Each document was screened against the final disease and mechanism dictionaries using regular expression-based pattern matching. To improve face validity, annotated records were manually inspected during dictionary refinement, and ambiguous terms or abbreviations were revised iteratively to reduce false-positive matches.

For each document, binary indicators were generated to denote whether a given disease or mechanism category was detected. Categories were not treated as mutually exclusive; therefore, a single document could be assigned to multiple disease categories and multiple mechanism categories. This multi-label approach reflected the fact that mitochondrial disease research frequently involves overlapping disease contexts and multiple mechanistic domains within the same abstract.

To improve interpretability in the main analyses, detailed disease and mechanism categories were additionally mapped into broader macro-groups. These macro-groups were used for visualization, disease–mechanism co-occurrence analysis and biological interpretation, while the full detailed dictionaries were retained for computational annotation and internal consistency checks. This two-level strategy allowed the analysis to preserve detailed molecular and disease-specific information while presenting the results in a more interpretable structure.

The annotation framework was therefore intended to capture explicit literature-level disease and mechanism signals rather than to provide full manual biomedical entity recognition. Dictionary-assigned labels were interpreted as corpus-level co-annotation indicators, and all downstream analyses were based on aggregated patterns rather than individual-document diagnostic classification.

#### Manual Validation of Dictionary-Based Annotation

After the dictionaries and matching rules had been finalized, a separate manual validation was performed to assess the accuracy of the regex-based dictionary annotation. A stratified validation sample of 200 title–abstract records was selected from the full corpus (n = 166,462) to provide broad representation across the three annotation layers (disease, mechanism, and drug) and their constituent categories. For each sampled record, dictionary-assigned labels were compared with manual review, and both incorrect assignments (false positives) and categories present in the reviewed text but missed by the dictionary (false negatives) were recorded.

The sampled records were manually reviewed, and uncertain or ambiguous cases were additionally reviewed by a second author and resolved by consensus [55]. For each annotation label, a true positive (TP) was defined as a dictionary-assigned label confirmed by manual review, a false positive (FP) as a dictionary-assigned label not supported by manual review, and a false negative (FN) as a label identified during manual review but missed by the dictionary. Label-level precision, recall, and F1-score were calculated as follows:

Precision=TP/(TP+FP);

Recall=TP/(TP+FN);

F1-score=2 × (Precision × Recall)/(Precision+Recall).

Micro-averaged metrics were calculated by pooling TP, FP, and FN counts across all labels within each annotation layer before metric calculation:

Micro-Precision=ΣTP/(ΣTP+ΣFP);

Micro-Recall=ΣTP/(ΣTP+ΣFN);

Micro-F1=2ΣTP/(2ΣTP+ΣFP+ΣFN).

Overall micro-averaged precision, recall, and F1-score were calculated by pooling TP, FP, and FN counts across all three annotation layers (disease, mechanism, and drug).

The macro-averaged F1-score was calculated as the unweighted arithmetic mean of the individual label-level F1-scores:

$$Macro\text{-}F1=\left({F1}_{1}+{F1}_{2}+\cdots +{F1}_{i}\right)/L$$

where L denotes the total number of assessable labels and ${F1}_{i}$ denotes the F1-score of the i-th label. Each assessable label contributed equally to Macro-F1, regardless of its frequency in the validation sample. Categories with no positive instances in the validation sample were excluded from the corresponding Macro-F1 calculation. The overall Macro-F1 was calculated across all 77 assessable labels and was not obtained by averaging the three annotation-layer Macro-F1 values.

### 4.7. Disease–Mechanism Co-Occurrence, Enrichment, and Pairwise Analyses

Disease–mechanism co-occurrence analysis was performed to examine how disease macro-groups and mitochondrial mechanism macro-groups were jointly represented within the corpus. For each disease–mechanism pair, the observed co-occurrence count was defined as the number of documents in which both the disease macro-group and the mechanism macro-group were detected. Because disease and mechanism categories were not mutually exclusive, a single document could contribute to more than one disease–mechanism pair.

Three complementary measures were calculated. First, the total corpus percentage was computed as the proportion of all documents in which a given disease–mechanism pair was detected. Second, the within-disease percentage was calculated as the proportion of documents within a given disease macro-group that also contained a specific mechanism macro-group. This measure was used to characterize disease-specific mitochondrial mechanism profiles. Third, lift was calculated to identify enriched disease–mechanism associations that occurred more frequently than expected under independence. Lift is defined as follows (Equation (1)):

(1) Lift=observed co-occurrence/expected co-occurrence under independence

where the expected co-occurrence was calculated as follows (Equation (2)):

(2) Expected co-occurrence=(number of documents in the disease macro-group × number of documents in the mechanism macro-group)/total number of documents

Lift values greater than 1 indicate that a disease–mechanism pair occurred more frequently than expected under independence. To reduce the influence of sparse or unstable associations, lift-based disease–mechanism visualizations were restricted to pairs with at least 500 co-occurring documents and lift values greater than 1. For the lift bubble plot, enriched pairs meeting these criteria were ranked by lift and document count, and the top 20 associations were displayed. The layered disease–mechanism network was constructed using the same document count and lift thresholds, with the top 30 enriched associations retained for visualization. Sensitivity checks were performed using alternative filtering criteria, including minimum co-occurrence thresholds of 250, 500, and 1000 documents and a stricter lift threshold of 1.25.

In addition to disease–mechanism co-occurrence, mechanism–mechanism and disease–disease pairwise co-occurrence analyses were performed to evaluate broader relational structures in the annotation framework. Pairwise co-occurrence summaries were ranked by co-occurring document counts, and the highest-ranking pairs were used to describe frequently co-occurring mechanism macro-groups and disease macro-groups.

### 4.8. Temporal Analyses of Dictionary-Based Disease and Mechanism Signals

Temporal analysis was performed to evaluate the changes in dictionary-based disease and mitochondrial mechanism signals across the study period. Publication years were grouped into three balanced periods: 2014–2017, 2018–2021, and 2022–2025. For each period, the number and percentage of documents assigned to each disease macro-group and mechanism macro-group were calculated. Percentages were computed using the total number of documents within the corresponding period as the denominator.

To summarize temporal shifts, absolute percentage-point changes were calculated between the earliest period and the most recent period. Positive values indicated increasing proportional representation in the recent literature, whereas negative values indicated decreasing proportional representation. This dictionary-based temporal analysis was used to provide an interpretable overview of emerging, persistent, and declining disease and mechanism signals before latent topic modeling.

### 4.9. NMF-Based Topic Modeling

Non-negative matrix factorization was used to identify interpretable latent thematic structures within the abstract corpus. Unlike dictionary-based annotation, which was designed to detect predefined disease and mechanism categories, NMF was applied as an unsupervised topic-modeling approach to extract recurring thematic patterns directly from article abstracts [27,45,46].

For NMF analysis, only abstracts were used. Compound expressions such as reactive oxygen species, oxidative stress, electron transport chain, oxidative phosphorylation, mitochondrial dynamics, mitochondrial dysfunction, mitochondrial membrane potential, cell death, endoplasmic reticulum stress, fatty acid oxidation, metabolic reprogramming, calcium signaling, mitochondrial biogenesis, mitochondrial quality control, and NAD+ were preserved as single lexical units where applicable.

Vocabulary filtering was performed to reduce noise from very rare or overly frequent terms. Terms were retained if they occurred at least 200 times in the corpus, appeared in at least 100 documents, and were present in no more than 40% of documents. A document–term matrix was constructed from the filtered abstract corpus. Term frequencies were log-transformed using log(1 + term frequency), and the matrix was weighted using inverse document frequency to generate the final TF–IDF representation for NMF.

Candidate NMF models were fitted using K = 10, 15, 20, 25, and 30 topics. Model selection was based on semantic coherence, topic exclusivity, and topic-size interpretability. Semantic coherence was evaluated using UMass coherence, where values closer to zero indicate stronger co-occurrence consistency among top topic terms. Topic exclusivity was calculated to assess the extent to which top terms were specific to individual topics. Topic-size distribution was additionally examined to identify potential topic fragmentation or very small topics.

A balance score was calculated as a weighted composite of semantic coherence and topic exclusivity, both of which were min–max scaled prior to combination. Because biological interpretability was prioritized, semantic coherence was assigned a higher weight than topic exclusivity. The balance score was defined as follows (Equation (3)):

(3) Balance score=0.70 × scaled semantic coherence+0.30 × scaled exclusivity

The candidate model with the highest balance score was selected as the final NMF solution, provided that the topic-size distribution remained interpretable and did not show excessive topic fragmentation. For reproducibility, candidate NMF models were fitted using fixed random seeds, a maximum of 300 iterations, and a convergence tolerance of 1 × 10^−4^.

After selecting the final topic number, each document was assigned to its dominant NMF topic based on the highest document–topic weight. For each topic, top-ranked terms and representative documents were extracted to support manual topic labeling. Final topic labels were assigned by jointly considering top terms, representative article titles and abstracts, and consistency with the disease–mechanism framework used in the dictionary-based analyses.

### 4.10. STM-Based Temporal Topic Modeling

Structural topic modeling was used as a covariate-aware complementary topic-modeling approach to examine temporal variations in topic prevalence across the abstract corpus. NMF and STM were not treated as competing models. Instead, NMF was used as the primary unsupervised method to define the main latent thematic structure of the corpus, whereas STM was used to evaluate how comparable thematic domains changed over time [23,26,28,51,56].

The STM analysis was performed using abstracts only. After preprocessing, vocabulary filtering, and removal of documents with insufficient remaining tokens, the final STM corpus consisted of 165,324 documents and 4662 terms. Terms occurring fewer than 500 times were removed, terms appearing in more than 35% of documents were excluded, and documents with fewer than 20 remaining tokens after trimming were removed.

The final STM model was fitted with K = 20 topics to maintain comparable thematic granularity with the final NMF solution. Publication year was modeled as a smooth temporal covariate to capture nonlinear temporal variation. Database source was included as an adjustment covariate in the primary database-adjusted STM specification to reduce the possibility that temporal topic patterns reflected changes in database coverage rather than thematic change. A year-only STM specification was additionally inspected as a robustness check.

To assess the robustness of the topic-number decision, K = 15 and K = 20 STM solutions were compared on a random subset of 40,000 documents drawn from the full preprocessed STM corpus. Both models were fitted using the same prevalence specification, initialization method, and maximum number of EM iterations. The models were compared using the mean semantic coherence, mean exclusivity, minimum and maximum topic prevalence, number of topics below 1% and 2% prevalence, and model lower bound. This comparison was used as a sensitivity check rather than as the primary basis for topic discovery.

For temporal interpretation, annual mean topic prevalence was calculated from document-level STM topic proportions. Topic-level year slopes were used as screening measures to identify increasing and decreasing topics, and temporal patterns were interpreted together with topic labels, top terms, and annual prevalence trajectories.

### 4.11. Exploratory Drug/Compound Annotation Layer

To explore translational and intervention-oriented signals within the corpus, an exploratory drug/compound annotation layer was added to the dictionary-based framework. The drug and bioactive compound dictionary was developed as a study-specific, corpus-informed lexicon using an iterative approach consistent with the disease and mechanism dictionary-development process. Candidate agents and compound groups were identified on the basis of their relevance to mitochondrial biology, pharmacological modulation, nutraceutical intervention, mitochondrial targeting, or experimental modulation of mitochondrial pathways, together with corpus-level inspection of recurrent drug- and compound-related expressions.

Candidate entries were expanded to include commonly used generic names, abbreviations, alternative names, and closely related lexical variants where appropriate. Entries were retained when they represented an identifiable pharmacological agent, bioactive compound, nutraceutical, mitochondria-targeted intervention, or experimentally relevant modulator with a clear relationship to mitochondrial biology. Broad pathway terms alone were not intended to constitute evidence of exposure to a specific drug or compound, and ambiguous expressions were refined or excluded when they produced recurrent context-independent matches. The final dictionary comprised 31 non-mutually exclusive drug/compound categories, including antidiabetic agents, anticancer targeted agents, mitochondrial antioxidants, NAD+ precursors, mTOR/autophagy modulators, senolytics, ferroptosis modulators, natural polyphenols, vitamins/micronutrients, and mitochondrial respiration/OXPHOS modulators.

Drug/compound annotation was performed on the same concatenated title–abstract field used for disease and mechanism annotation. For each document, binary indicators were generated to denote whether each drug/compound category was detected. The drug/compound annotation layer was included in the manual validation procedure described in Section Manual Validation of Dictionary-Based Annotation of Section 4.6, in which its precision, recall, and F1 performance were evaluated alongside the disease and mechanism annotation layers.

Co-occurrence analysis was performed between drug/compound categories and mitochondrial mechanism macro-groups to provide an exploratory translational context. Because this layer was intended to support biological interpretation rather than define the main disease–mechanism structure, the findings were interpreted as literature co-annotation signals rather than evidence of therapeutic efficacy.

### 4.12. Visualization, Software, and Reproducibility

Visualization strategies were selected according to the analytical layer. Bar plots were used to summarize the disease and mechanism macro-group frequencies. Heatmaps were used to display disease–mechanism and drug/compound–mechanism co-occurrence patterns based on within-category percentages. Bubble plots were used to visualize enriched disease–mechanism associations according to lift values, with bubble size representing the document count. Network visualizations were used to represent enriched disease–mechanism associations and mechanism–mechanism co-occurrence structures. Temporal bar plots were used to summarize dictionary-based changes across periods. Topic prevalence plots and temporal trajectory plots were used to present NMF and STM outputs.

All data processing, text preprocessing, dictionary-based annotation, topic modeling, and visualization procedures were performed using R software version 4.5.0 (R Core Team, 2025) within the RStudio Desktop-integrated development environment version 2025.05.1+513, Mariposa Orchid (Posit Team, 2025) [57,58]. Data management and wrangling were performed using dplyr, tidyr, readr, and readxl. Text processing and string operations were performed using stringr, stringi, tidytext, and quanteda. Regular expression-based disease, mechanism, and drug/compound annotations were implemented using the final dictionaries developed for this study. Sparse document–term matrices and TF–IDF-weighted representations were prepared using text-mining and matrix-processing tools available.

NMF-based topic modeling was performed using R packages for non-negative matrix factorization and topic-model evaluation. Candidate NMF models were compared using semantic coherence, topic exclusivity, topic-size distribution, and a combined balance score. STM-based temporal topic modeling was performed using the stm package, with the publication year modeled as a smooth temporal covariate and database source included as an adjustment covariate in the primary specification. Visualization was performed using ggplot2, with network-based visualizations generated using igraph and ggraph. Tables and analytical outputs were exported using spreadsheet-writing tools.

Final dictionaries, macro-group mappings, and processed analytical outputs can be made available upon reasonable request to support transparency and reproducibility.

## 5. Conclusions

This study mapped mitochondrial bioenergetics and signaling research in disease contexts from 2014 to 2025 using an integrated text-mining framework combining dictionary-based annotation and topic modeling. The literature was organized around a central mechanistic backbone involving ROS/redox biology, cell death pathways, bioenergetics/OXPHOS, mitochondrial dysfunction/homeostasis, and mitochondrial quality control. Disease–mechanism enrichment analysis revealed biologically coherent signatures, including mtDNA/genome integrity in primary mitochondrial and genetic disease, inflammation/innate immunity in inflammatory and immune-mediated disease, metabolic regulation in liver and cardiometabolic disease, stress adaptation in aging-related disease, and cell death/ROS-related mechanisms in toxicological or drug-induced injury.

NMF identified a 20-topic thematic structure covering both broad mitochondrial themes and disease-specific domains, while STM showed that recent literature increasingly emphasizes mitochondrial dysfunction, immune-inflammatory signaling, omics-based prognostic signatures, delivery systems for clinical application, and cancer progression/resistance. In contrast, earlier relative emphasis on apoptosis, mtDNA/genetic disorders, calcium signaling and compound-screening topics has decreased.

Overall, the findings suggest that mitochondrial disease research is shifting toward an integrated, application-oriented framework in which mitochondria are positioned as bioenergetic, signaling, immune-regulatory and therapeutic-response hubs across diverse disease contexts. Future work combining full-text mining, cross-lingual corpora and longitudinal validation could further refine the mechanistic and translational trajectories identified here.

## Figures and Tables

**Table 1:** Manual validation performance of the dictionary-based annotation framework.

Annotation Layer	TP	FP	FN	Micro-Precision	Micro-Recall	Micro-F1	Macro-F1
Disease	338	26	25	0.929	0.931	0.930	0.916
Mechanism	679	15	33	0.978	0.954	0.966	0.954
Drug	79	10	6	0.888	0.929	0.908	0.906
Overall	1096	51	64	0.956	0.945	0.950	0.926

TP, true positive; FP, false positive; FN, false negative. Micro-Precision, Micro-Recall, and Micro-F1 were calculated by pooling TP, FP, and FN counts across all labels within each annotation layer. Overall micro-averaged metrics were calculated by pooling TP, FP, and FN counts across the disease, mechanism, and drug annotation layers. Macro-F1 represents the unweighted arithmetic mean of the individual label-level F1-scores among assessable categories, thereby assigning equal weight to each assessable label regardless of its frequency. The overall Macro-F1 was calculated across all 77 assessable labels and was not derived by averaging the three annotation-layer Macro-F1 values. A total of 2 of the 31 drug categories were not represented in the validation sample and therefore could not be independently evaluated; these categories were excluded from the Macro-F1 calculation.

**Table 2:** Summary of NMF model selection metrics.

K	Mean UMass Coherence	Mean Exclusivity	Min Topic %	Max Topic %	Topics < 1%	Balance Score
**10**	−1.9151	0.7879	3.21	22.46	0	0.3126
**15**	−1.8669	0.7623	2.22	19.75	0	0.6702
**20**	−1.8390	0.7185	0.71	20.58	1	0.7891
**25**	−1.8607	0.7324	0.84	10.81	2	0.6353
**30**	−1.9165	0.6892	0.80	14.80	3	0.0000

Candidate NMF models were evaluated using UMass semantic coherence, topic exclusivity, topic-size distribution, and a combined balance score. K = 20 was retained because it achieved the highest balance score and the strongest mean semantic coherence while maintaining an interpretable topic-size distribution.

**Table 3:** Final NMF-derived topic labels and thematic interpretation.

Topic	Documents (%)	Main Disease/Mechanism Axis	Representative Top Terms
**Topic 1**	34,254 (20.58%)	Aging/senescence/mitochondrial homeostasis	aging, cellular, homeostasis, senescence, molecular, metabolic
**Topic 2**	15,548 (9.34%)	Reproductive and developmental mitochondrial biology	age, exposure, male, female, blood, plasma
**Topic 3**	15,616 (9.38%)	Cancer metabolism/bioenergetics	glycolysis, metabolism, cancer, tumor, oxidative phosphorylation, ATP
**Topic 4**	12,774 (7.67%)	mtDNA/mitochondrial genetics	mtDNA, mutations, variants, sequencing, genome, copy
**Topic 5**	10,331 (6.21%)	Translational cancer therapy/drug delivery	tumor, nanoparticles, delivery, drug, PDT, targeting
**Topic 6**	10,524 (6.32%)	Anticancer screening/cytotoxicity	compounds, anticancer, IC50, cytotoxicity, breast, A549
**Topic 7**	11,939 (7.17%)	Neurodegeneration/neuronal injury	brain, neurons, neurodegenerative, cognitive, Alzheimer, Parkinson
**Topic 8**	9200 (5.53%)	Apoptosis/regulated cell death	caspase, BCL, apoptosis, BAX, cytochrome, p53
**Topic 9**	5865 (3.52%)	Omics/prognosis/immune signatures	prognostic, enrichment, immune, differentially expressed, DEGs, risk
**Topic 10**	6928 (4.16%)	Inflammation/innate immunity	inflammatory, NLRP3, macrophages, inflammasome, LPS, cytokines
**Topic 11**	4621 (2.78%)	Experimental validation methods	Western blot, staining, assay, flow cytometry, PCR, microscopy
**Topic 12**	4813 (2.89%)	Mitophagy/mitochondrial quality control	mitophagy, autophagy, Parkin, PINK1, fission, LC3
**Topic 13**	6209 (3.73%)	Cardiovascular injury/ischemia–reperfusion	cardiac, myocardial, reperfusion, ischemia, injury, cardiomyocytes
**Topic 14**	1963 (1.18%)	Mitochondrial biogenesis/nutrient-sensing signaling	PGC, AMPK, PPAR, mitochondrial biogenesis, receptor, coactivator
**Topic 15**	2880 (1.73%)	Ferroptosis/oxidative lipid damage	ferroptosis, iron, GPX4, glutathione, lipid peroxidation, NRF2
**Topic 16**	3429 (2.06%)	Cardiometabolic disease/adipose metabolism	obesity, insulin, adipose, high-fat diet, glucose, diabetes
**Topic 17**	3066 (1.84%)	Liver disease/steatotic liver disease	liver, hepatic, NAFLD, steatosis, NASH, fibrosis
**Topic 18**	3029 (1.82%)	Muscle/exercise/aging	muscle, skeletal, exercise, sarcopenia, atrophy, training
**Topic 19**	2298 (1.38%)	Kidney disease/renal injury	renal, kidney, AKI, tubular injury, CKD, fibrosis
**Topic 20**	1175 (0.71%)	Non-coding RNA regulation	miR, miRNAs, microRNA, luciferase, RNA, target

Final NMF topics were assigned by jointly considering representative top terms, representative documents, and consistency with the disease–mechanism framework. Document counts and percentages indicate the number and proportion of abstracts assigned to each dominant NMF topic.

**Table 4:** STM topics showing the greatest temporal changes.

Topic	Proposed Label	Prevalence (%)	Year Slope	Trend
**Topic 10**	Apoptosis, autophagy and BCL-2/BAX-mediated cell death	4.87	−0.00524	Decreasing
**Topic 6**	Gene-expression, transcriptomic and prognostic signatures	4.50	+0.00427	Increasing
**Topic 18**	Review-oriented neurodegeneration, mitochondrial dysfunction, and therapeutic perspectives	11.50	+0.00373	Increasing
**Topic 7**	Inflammation, immune response and infection-related mitochondrial dysfunction	4.55	+0.00331	Increasing
**Topic 4**	Nanoparticle-based drug delivery, imaging and photodynamic/photothermal therapy	4.89	+0.00316	Increasing
**Topic 12**	DNA sequence/species-level genetic distribution and comparative mtDNA sequence content	4.07	−0.00307	Decreasing
**Topic 5**	mtDNA mutations, variants, and mitochondrial genetic disorders	4.66	−0.00254	Decreasing
**Topic 2**	Anticancer compounds and cytotoxicity screening	6.27	−0.00243	Decreasing
**Topic 3**	Calcium signaling, membrane homeostasis, and NLRP3/pyroptosis	5.42	−0.00160	Decreasing
**Topic 11**	Cancer progression, resistance, and metastasis	5.72	+0.00111	Increasing

## Data Availability

The data presented in this study are available on request from the corresponding author due to legal restrictions (third-party licensing agreements of commercial bibliographic databases).

## Abbreviations

The following abbreviations are used in this manuscript:

NMF	Non-negative matrix factorization
STM	Structural topic modeling
OXPHOS	Oxidative phosphorylation
ROS	Reactive oxygen species
TF–IDF	Term frequency–inverse document frequency
mtDNA	Mitochondrial DNA
ATP	Adenosine triphosphate
EM	Expectation–maximization
AMPK	AMP-activated protein kinase
mTOR	Mechanistic target of rapamycin
NLRP3	NLR family pyrin domain-containing protein 3
PINK1	PTEN-induced kinase 1
GPX4	Glutathione peroxidase 4
NRF2	Nuclear factor erythroid 2-related factor 2
LC3	Microtubule-associated protein 1A/1B-light chain 3
PGC	Peroxisome proliferator-activated receptor gamma coactivator
PPAR	Peroxisome proliferator-activated receptor
BCL-2	B-cell lymphoma 2
BAX	BCL-2-associated X protein
AKI	Acute kidney injury
CKD	Chronic kidney disease
NAFLD	Non-alcoholic fatty liver disease
NASH	Non-alcoholic steatohepatitis
DEGs	Differentially expressed genes
LPS	Lipopolysaccharide
PDT	Photodynamic therapy
IC50	Half-maximal inhibitory concentration
FREX	Frequency and exclusivity
UMass	University of Massachusetts (coherence metric)

## References

[1] Zong Y., Li H., Liao P., Chen L., Pan Y., Zheng Y., Zhang C., Liu D., Zheng M., Gao J. (2024). Mitochondrial dysfunction: Mechanisms and advances in therapy. Signal Transduct. Target. Ther..

[2] Selim N.A., Wojtovich A.P. (2025). Mitochondrial membrane potential and compartmentalized signaling: Calcium, ROS, and beyond. Redox Biol..

[3] Clemente-Suárez V.J., Redondo-Flórez L., Beltrán-Velasco A.I., Ramos-Campo D.J., Belinchón-deMiguel P., Martinez-Guardado I., Dalamitros A.A., Yáñez-Sepúlveda R., Martín-Rodríguez A., Tornero-Aguilera J.F. (2023). Mitochondria and brain disease: A comprehensive review of pathological mechanisms and therapeutic opportunities. Biomedicines.

[4] Yu J., Huang Y., Qin Y., Zhu J., Zhao T., Wu H., Ye X., Qin X., Li S., Chen Y. (2025). Deciphering mitochondria: Unveiling their roles in mechanosensing and mechanotransduction. Research.

[5] Harrington J.S., Ryter S.W., Plataki M., Price D.R., Choi A.M.K. (2023). Mitochondria in health, disease, and aging. Physiol. Rev..

[6] Tan J.X., Finkel T. (2020). Mitochondria as intracellular signaling platforms in health and disease. J. Cell Biol..

[7] Suomalainen A., Nunnari J. (2024). Mitochondria at the crossroads of health and disease. Cell.

[8] Collier J.J., Oláhová M., McWilliams T.G., Taylor R.W. (2023). Mitochondrial signalling and homeostasis: From cell biology to neurological disease. Trends Neurosci..

[9] Ronayne C.T., Latorre-Muro P. (2024). Navigating the landscape of mitochondrial-ER communication in health and disease. Front. Mol. Biosci..

[10] Andrieux P., Chevillard C., Cunha-Neto E., Nunes J.P.S. (2021). Mitochondria as a cellular hub in infection and inflammation. Int. J. Mol. Sci..

[11] Nunnari J., Suomalainen A. (2012). Mitochondria: In sickness and in health. Cell.

[12] Chen W., Zhao H., Li Y. (2023). Mitochondrial dynamics in health and disease: Mechanisms and potential targets. Signal Transduct. Target. Ther..

[13] El Wakil A., Devos P., Abdelmegeed H., Kamel A. (2025). Mitochondria in cancer: A comprehensive review, bibliometric analysis, and future perspectives. Discov. Oncol..

[14] Yang H.-M. (2025). Mitochondrial dysfunction in cardiovascular diseases. Int. J. Mol. Sci..

[15] Yang H.-M. (2025). Mitochondrial dysfunction in neurodegenerative diseases. Cells.

[16] Pliouta L., Lampsas S., Kountouri A., Korakas E., Thymis J., Kassi E., Oikonomou E., Ikonomidis I., Lambadiari V. (2025). Mitochondrial dysfunction in the development and progression of cardiometabolic diseases: A narrative review. J. Clin. Med..

[17] Shi S., Zhang B., Li Y., Xu X., Lv J., Jia Q., Chai R., Xue W., Li Y., Wang Y. (2022). Mitochondrial dysfunction: An emerging link in the pathophysiology of cardiorenal syndrome. Front. Cardiovasc. Med..

[18] Grünewald A., Kumar K.R., Sue C.M. (2019). New insights into the complex role of mitochondria in Parkinson’s disease. Prog. Neurobiol..

[19] Du H., Xu T., Yu S., Wu S., Zhang J. (2025). Mitochondrial metabolism and cancer therapeutic innovation. Signal Transduct. Target. Ther..

[20] Bartman S., Coppotelli G., Ross J.M. (2024). Mitochondrial dysfunction: A key player in brain aging and diseases. Curr. Issues Mol. Biol..

[21] Son J.M., Lee C. (2021). Aging: All roads lead to mitochondria. Semin. Cell Dev. Biol..

[22] Radhakrishnan S., Erbis S., Isaacs J.A., Kamarthi S. (2017). Novel keyword co-occurrence network-based methods to foster systematic reviews of scientific literature. PLoS ONE.

[23] Tan Y., Liu M., Zhou X., Gao T., Fang J., Wang S., Chen S. (2024). Mapping the mitochondrial landscape in T2DM: Key findings from 2003–2023. Front. Endocrinol..

[24] Hu S., Yang Z., Bao K., Hou W., Qin Y., Wu C., Wang Q., Luo X., Luo L. (2025). Mitochondrial dynamics in neurodegenerative diseases: Research hotspots and trends from 2005 to 2025. Ageing Res. Rev..

[25] Pang J., Zhang Y., Tian Y., Cao X., Tao X., Sun C., Cao Z. (2026). Mapping the scientific landscape of mitochondria-associated membranes: A bibliometric insight into emerging frontiers in aging and diseases. Int. J. Surg..

[26] Hannigan T.R., Haans R.F.J., Vakili K., Tchalian H., Glaser V.L., Wang M.S., Kaplan S., Jennings P.D. (2019). Topic modeling in management research: Rendering new theory from textual data. Acad. Manag. Ann..

[27] Liu L., Tang L., Dong W., Yao S., Zhou W. (2016). An overview of topic modeling and its current applications in bioinformatics. SpringerPlus.

[28] Churchill R., Singh L. (2022). The evolution of topic modeling. ACM Comput. Surv..

[29] Picard M., Wallace D.C., Burelle Y. (2016). The rise of mitochondria in medicine. Mitochondrion.

[30] Nolfi-Donegan D., Braganza A., Shiva S. (2020). Mitochondrial electron transport chain: Oxidative phosphorylation, oxidant production, and methods of measurement. Redox Biol..

[31] Tilokani L., Nagashima S., Paupe V., Prudent J. (2018). Mitochondrial dynamics: Overview of molecular mechanisms. Essays Biochem..

[32] Mihaylova V., Kovacheva E., Gevezova M., Sarafian V., Kazakova M. (2026). Mitochondria as an integrative hub of cellular homeostasis and stress response. Int. J. Mol. Sci..

[33] Wallace D.C. (2012). Mitochondria and cancer. Nat. Rev. Cancer.

[34] Porporato P.E., Filigheddu N., Pedro J.M.B.-S., Kroemer G., Galluzzi L. (2018). Mitochondrial metabolism and cancer. Cell Res..

[35] Faubert B., Solmonson A., DeBerardinis R.J. (2020). Metabolic reprogramming and cancer progression. Science.

[36] Sivitz W.I., Yorek M.A. (2009). Mitochondrial dysfunction in diabetes: From molecular mechanisms to functional significance and therapeutic opportunities. Antioxid. Redox Signal..

[37] Zhou B., Tian R. (2018). Mitochondrial dysfunction in pathophysiology of heart failure. J. Clin. Investig..

[38] Green D.R., Galluzzi L., Kroemer G. (2011). Mitochondria and the autophagy–inflammation–cell death axis in organismal aging. Science.

[39] Chen G., Kroemer G., Kepp O. (2020). Mitophagy: An emerging role in aging and age-associated diseases. Front. Cell Dev. Biol..

[40] Li A., Gao M., Liu B., Qin Y., Chen L., Liu H., Wu H., Gong G. (2022). Mitochondrial autophagy: Molecular mechanisms and implications for cardiovascular disease. Cell Death Dis..

[41] Narendra D.P., Youle R.J. (2024). The role of PINK1–Parkin in mitochondrial quality control. Nat. Cell Biol..

[42] West A.P., Shadel G.S. (2017). Mitochondrial DNA in innate immune responses and inflammatory pathology. Nat. Rev. Immunol..

[43] Zhu X., Chen Z., Shen W., Huang G., Sedivy J.M., Wang H., Ju Z. (2021). Inflammation, epigenetics, and metabolism converge to cell senescence and ageing: The regulation and intervention. Signal Transduct. Target. Ther..

[44] Guo J., Huang X., Dou L., Yan M., Shen T., Tang W., Li J. (2022). Aging and aging-related diseases: From molecular mechanisms to interventions and treatments. Signal Transduct. Target. Ther..

[45] Devarajan K. (2008). Nonnegative matrix factorization: An analytical and interpretive tool in computational biology. PLoS Comput. Biol..

[46] Pascual-Montano A., Carmona-Saez P., Chagoyen M., Tirado F., Carazo J.M., Pascual-Marqui R.D. (2006). bioNMF: A versatile tool for non-negative matrix factorization in biology. BMC Bioinform..

[47] Stockwell B.R., Friedmann Angeli J.P., Bayir H., Bush A.I., Conrad M., Dixon S.J., Fulda S., Gascón S., Hatzios S.K., Kagan V.E. (2017). Ferroptosis: A regulated cell death nexus linking metabolism, redox biology, and disease. Cell.

[48] Feng F., He S., Li X., He J., Luo L. (2024). Mitochondria-mediated ferroptosis in diseases therapy: From molecular mechanisms to implications. Aging Dis..

[49] Roberts M.E., Stewart B.M., Tingley D. (2019). stm: An R package for structural topic models. J. Stat. Softw..

[50] Xu J., Shamul J.G., Kwizera E.A., He X. (2022). Recent advancements in mitochondria-targeted nanoparticle drug delivery for cancer therapy. Nanomaterials.

[51] Vanhala M., Lu C., Peltonen J., Sundqvist S., Nummenmaa J., Järvelin K. (2020). The usage of large data sets in online consumer behaviour: A bibliometric and computational text-mining–driven analysis of previous research. J. Bus. Res..

[52] Elsevier (2004). Scopus: Abstract and Citation Database. https://www.scopus.com.

[53] Clarivate (1997). Web of Science: Citation Indexing and Search Platform. https://www.webofscience.com.

[54] National Center for Biotechnology Information (NCBI) (1996). PubMed: Biomedical Literature Database. National Library of Medicine. https://pubmed.ncbi.nlm.nih.gov.

[55] Yonar H., Beşoluk F.Ç., Yonar A. (2026). Thematic mapping and sustainability-oriented analysis of veterinary science research in Türkiye: A text mining and hybrid hierarchical clustering approach (1980–2024). Kafkas Univ. Vet. Fak. Derg..

[56] Yonar H., Beşoluk F.Ç. (2026). A multi-field text mining and topic modelling approach to the Potato Research Journal (1970–2024). Potato Res..

[57] R Core Team (2025). R: A Language and Environment for Statistical Computing.

[58] Posit Team RStudio: *Integrated Development Environment for R*, RStudio Desktop Version 2025.05.1+513, Mariposa Orchid; Posit Software, PBC: Boston, MA, USA, 2025. https://posit.co/.

